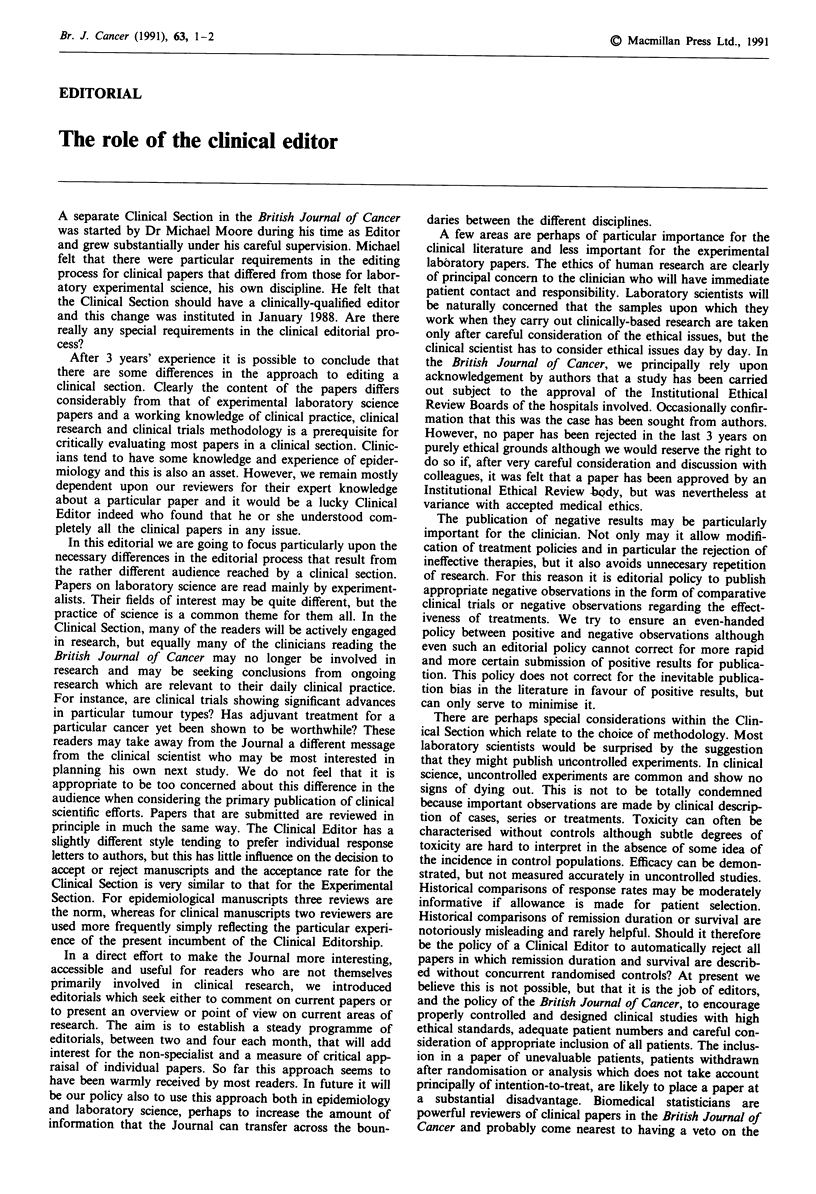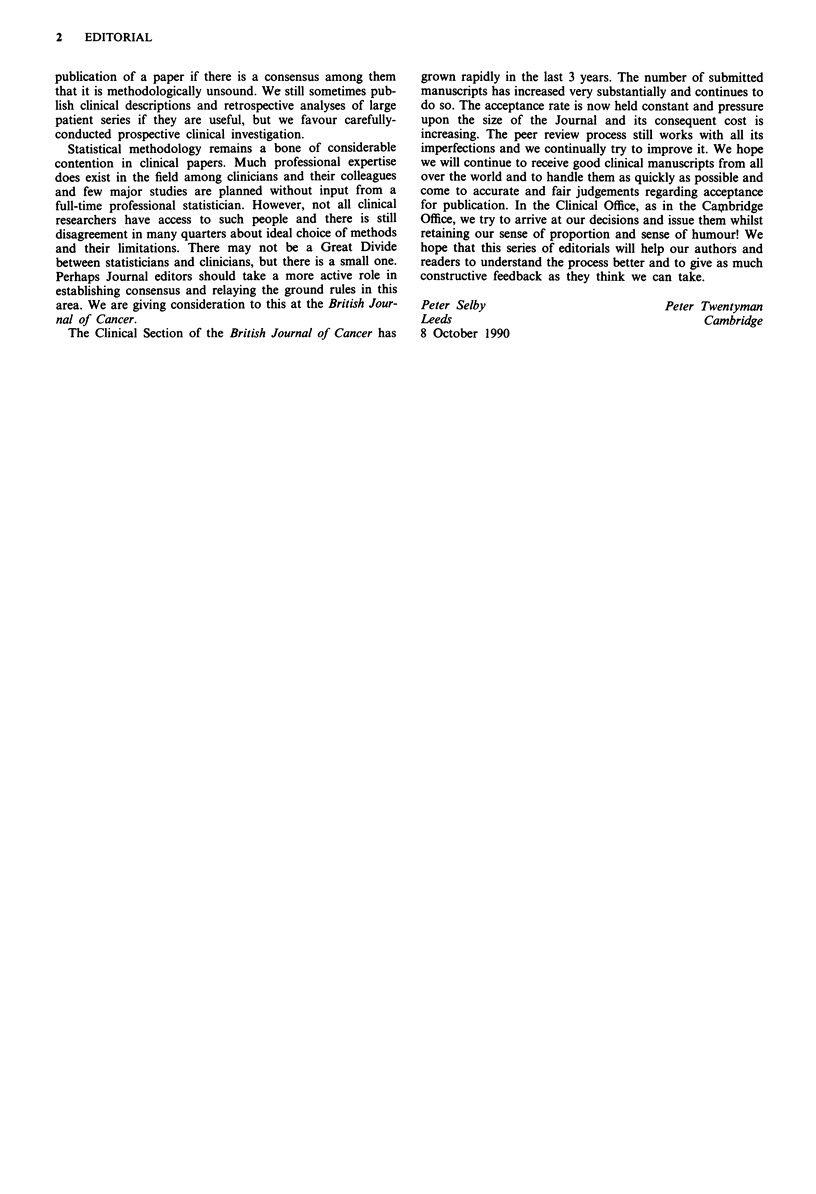# The role of the clinical editor.

**DOI:** 10.1038/bjc.1991.1

**Published:** 1991-01

**Authors:** P. Selby, P. Twentyman


Br. J. Cancer (1991), 63, 1–2
© Macmillan Press Ltd., 1991

EDITORIAL

### The role of the clinical editor

A separate Clinical Section in the *British Journal of Cancer* was started by Dr Michael Moore during his time as Editor and grew substantially under his careful supervision. Michael felt that there were particular requirements in the editing process for clinical papers that differed from those for laboratory experimental science, his own discipline. He felt that the Clinical Section should have a clinically-qualified editor and this change was instituted in January 1988. Are there really any special requirements in the clinical editorial process?

After 3 years' experience it is possible to conclude that there are some differences in the approach to editing a clinical section. Clearly the content of the papers differs considerably from that of experimental laboratory science papers and a working knowledge of clinical practice, clinical research and clinical trials methodology is a prerequisite for critically evaluating most papers in a clinical section. Clinicians tend to have some knowledge and experience of epidermiology and this is also an asset. However, we remain mostly dependent upon our reviewers for their expert knowledge about a particular paper and it would be a lucky Clinical Editor indeed who found that he or she understood completely all the clinical papers in any issue.

In this editorial we are going to focus particularly upon the necessary differences in the editorial process that result from the rather different audience reached by a clinical section. Papers on laboratory science are read mainly by experimentalists. Their fields of interest may be quite different, but the practice of science is a common theme for them all. In the Clinical Section, many of the readers will be actively engaged in research, but equally many of the clinicians reading the *British Journal of Cancer* may no longer be involved in research and may be seeking conclusions from ongoing research which are relevant to their daily clinical practice. For instance, are clinical trials showing significant advances in particular tumour types? Has adjuvant treatment for a particular cancer yet been shown to be worthwhile? These readers may take away from the Journal a different message from the clinical scientist who may be most interested in planning his own next study. We do not feel that it is appropriate to be too concerned about this difference in the audience when considering the primary publication of clinical scientific efforts. Papers that are submitted are reviewed in principle in much the same way. The Clinical Editor has a slightly different style tending to prefer individual response letters to authors, but this has little influence on the decision to accept or reject manuscripts and the acceptance rate for the Clinical Section is very similar to that for the Experimental Section. For epidemiological manuscripts three reviews are the norm, whereas for clinical manuscripts two reviewers are used more frequently simply reflecting the particular experience of the present incumbent of the Clinical Editorship.

In a direct effort to make the Journal more interesting, accessible and useful for readers who are not themselves primarily involved in clinical research, we introduced editorials which seek either to comment on current papers or to present an overview or point of view on current areas of research. The aim is to establish a steady programme of editorials, between two and four each month, that will add interest for the non-specialist and a measure of critical appraisal of individual papers. So far this approach seems to have been warmly received by most readers. In future it will be our policy also to use this approach both in epidemiology and laboratory science, perhaps to increase the amount of information that the Journal can transfer across the boundaries between the different disciplines.

A few areas are perhaps of particular importance for the clinical literature and less important for the experimental laboratory papers. The ethics of human research are clearly of principal concern to the clinician who will have immediate patient contact and responsibility. Laboratory scientists will be naturally concerned that the samples upon which they work when they carry out clinically-based research are taken only after careful consideration of the ethical issues, but the clinical scientist has to consider ethical issues day by day. In the *British Journal of Cancer*, we principally rely upon acknowledgement by authors that a study has been carried out subject to the approval of the Institutional Ethical Review Boards of the hospitals involved. Occasionally confirmation that this was the case has been sought from authors. However, no paper has been rejected in the last 3 years on purely ethical grounds although we would reserve the right to do so if, after very careful consideration and discussion with colleagues, it was felt that a paper has been approved by an Institutional Ethical Review body, but was nevertheless at variance with accepted medical ethics.

The publication of negative results may be particularly important for the clinician. Not only may it allow modification of treatment policies and in particular the rejection of ineffective therapies, but it also avoids unnecesary repetition of research. For this reason it is editorial policy to publish appropriate negative observations in the form of comparative clinical trials or negative observations regarding the effectiveness of treatments. We try to ensure an even-handed policy between positive and negative observations although even such an editorial policy cannot correct for more rapid and more certain submission of positive results for publication. This policy does not correct for the inevitable publication bias in the literature in favour of positive results, but can only serve to minimise it.

There are perhaps special considerations within the Clinical Section which relate to the choice of methodology. Most laboratory scientists would be surprised by the suggestion that they might publish uncontrolled experiments. In clinical science, uncontrolled experiments are common and show no signs of dying out. This is not to be totally condemned because important observations are made by clinical description of cases, series or treatments. Toxicity can often be characterised without controls although subtle degrees of toxicity are hard to interpret in the absence of some idea of the incidence in control populations. Efficacy can be demonstrated, but not measured accurately in uncontrolled studies. Historical comparisons of response rates may be moderately informative if allowance is made for patient selection. Historical comparisons of remission duration or survival are notoriously misleading and rarely helpful. Should it therefore be the policy of a Clinical Editor to automatically reject all papers in which remission duration and survival are described without concurrent randomised controls? At present we believe this is not possible, but that it is the job of editors, and the policy of the *British Journal of Cancer*, to encourage properly controlled and designed clinical studies with high ethical standards, adequate patient numbers and careful consideration of appropriate inclusion of all patients. The inclusion in a paper of unevaluable patients, patients withdrawn after randomisation or analysis which does not take account principally of intention-to-treat, are likely to place a paper at a substantial disadvantage. Biomedical statisticians are powerful reviewers of clinical papers in the *British Journal of Cancer* and probably come nearest to having a veto on the publication of a paper if there is a consensus among them that it is methodologically unsound. We still sometimes publish clinical descriptions and retrospective analyses of large patient series if they are useful, but we favour carefully-conducted prospective clinical investigation.

Statistical methodology remains a bone of considerable contention in clinical papers. Much professional expertise does exist in the field among clinicians and their colleagues and few major studies are planned without input from a full-time professional statistician. However, not all clinical researchers have access to such people and there is still disagreement in many quarters about ideal choice of methods and their limitations. There may not be a Great Divide between statisticians and clinicians, but there is a small one. Perhaps Journal editors should take a more active role in establishing consensus and relaying the ground rules in this area. We are giving consideration to this at the *British Journal of Cancer*.

The Clinical Section of the *British Journal of Cancer* has grown rapidly in the last 3 years. The number of submitted manuscripts has increased very substantially and continues to do so. The acceptance rate is now held constant and pressure upon the size of the Journal and its consequent cost is increasing. The peer review process still works with all its imperfections and we continually try to improve it. We hope we will continue to receive good clinical manuscripts from all over the world and to handle them as quickly as possible and come to accurate and fair judgements regarding acceptance for publication. In the Clinical Office, as in the Cambridge Office, we try to arrive at our decisions and issue them whilst retaining our sense of proportion and sense of humour! We hope that this series of editorials will help our authors and readers to understand the process better and to give as much constructive feedback as they think we can take.

*Peter Selby*
*Leeds*

*Peter Twentyman*
*Cambridge*

8 October 1990